# *Clostridium cadaveris* Osteomyelitis: an Unusual Pathogen which Highlights the Importance of Deep Tissue Sampling in Chronic Osteomyelitis

**DOI:** 10.7150/jbji.43801

**Published:** 2020-03-30

**Authors:** Ruth Alexandra Corrigan, Jose Lomas-Cabeza, David Stubbs, Martin McNally

**Affiliations:** 1 Bone Infection Unit, Nuffield Orthopaedic Centre, Oxford University Hospitals NHS Foundation Trust, Windmill Road, Oxford, OX3 7HE, UK.; 2 Nuffield Department of Clinical Laboratory Sciences, Radcliffe Department of Medicine, John Radcliffe Hospital, Headley Way, Oxford, OX3 9DU, UK.

**Keywords:** Chronic osteomyelitis, intra-operative sampling, *Clostridium cadaveris*

## Abstract

*Clostridium cadaveris*, named following its identification in human corpses, is an unusual pathogen. We report the first case of *C. cadaveris* osteomyelitis. This case highlights the importance of deep tissue sampling and appropriate culture to correctly identify causative pathogens and guide targeted antimicrobial therapy in difficult-to-treat infections like chronic osteomyelitis.

## Background

The genus Clostridium contains over 220 obligate anaerobic (occasionally aerotolerant species) most of which are considered clinically insignificant environmental bacteria. *C. cadaveris* is usually a non-pathogenic and non-toxin producing, enteric, gas-forming anaerobe. First described by Klein in 1899 as the most prominent bacteria present during human decomposition 1, it is found in both the human gut and the environment.

Bacteraemia with Clostridia is rare, calculated to be responsible for 0.5%-2% of all positive anaerobic blood cultures in one study 2. Infections are most often gastrointestinal in origin 2,3 and affect those with predisposing immunosuppression (malignancy, diabetes, alcohol abuse, steroids, chemotherapy) 3-6. There are approximately 30 cases of Clostridial osteomyelitis published to date (Table 1 7-25). Of note, since 2016, Clostridioides difficile (previously Clostridium difficile) is considered a different genus based on phenotypic, chemotaxonomic and phylogenetic analysis 26 and so is not considered further here. Over 30 cases of clostridial septic arthritis have also recently been summarised 27. Osteomyelitis due to clostridial species usually follows trauma 14 or open fractures 7,8,11,12,15-18, some obviously contaminated with soil 12,15,17 or sewerage 8, 11. In non-traumatic cases hosts may be immunocompromised by lymphoma 13,22 or diabetes 10, though sometimes there is neither a clearly identifiable source nor host risk factor 9. Nine of these cases have been reported in the last five years which likely reflects laboratory advances in diagnostic capabilities including improved anaerobic culture techniques and the increasing availability of MALDI-TOF mass spectrometry and genetic sequencing for exact species determination.

Although there are some reports of non-orthopaedic infections in immunocompetent individuals 28 this is the first case report of *C. cadaveris* osteomyelitis in either immunocompromised or healthy individuals.

Chronic osteomyelitis can be a notoriously difficult infection to diagnose and treat. A high clinical index of suspicion, appropriate imaging, thorough debridement and long-term targeted antibiotic therapy is widely considered the best management strategy. Thorough tissue sampling and appropriate culture is essential to identify causative organisms to permit targeted antibiotic therapy and effective treatment 29,30.

### Case presentation

A 32 year old man presented to us with a 12 month history of infection around his lower right tibia and ankle. There was no history of fracture; rather, the infection was attributed to a soft tissue injury sustained after impact with a concrete bollard whilst running. Before presentation to us he had undergone incision and drainage of a large volume of pus as well as windowing of the tibia and two bony debridement procedures, the last 6 months previously. A wound swab taken at the time of injury had grown a fully sensitive Group A haemolytic streptococcus, and a single intraoperative sample from the right distal tibia taken at the time of the last debridement had grown *Staphylococcus aureus* (resistant to erythromycin). Blood cultures had remained negative throughout. He had completed a short course of oral clindamycin 300mg QDS (5 days) and oral flucloxacillin 500mg QDS prescribed by his GP at the time of initial presentation, as well as a second short course of oral flucloxacillin and two short courses of oral amoxicillin immediately after each of his surgical procedures. He was a smoker, but had no other significant medical co-morbidities.

**Figure 1:**
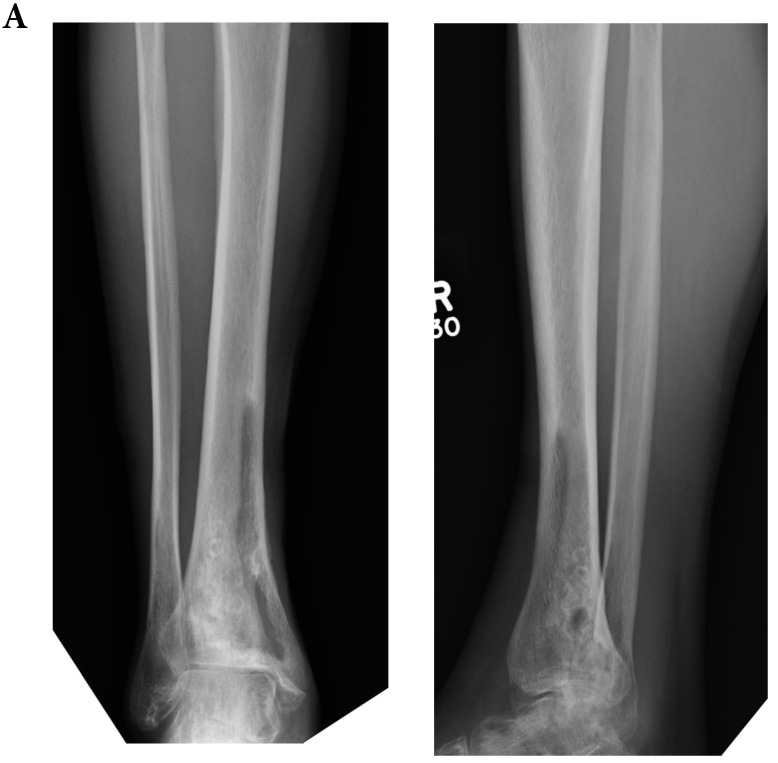

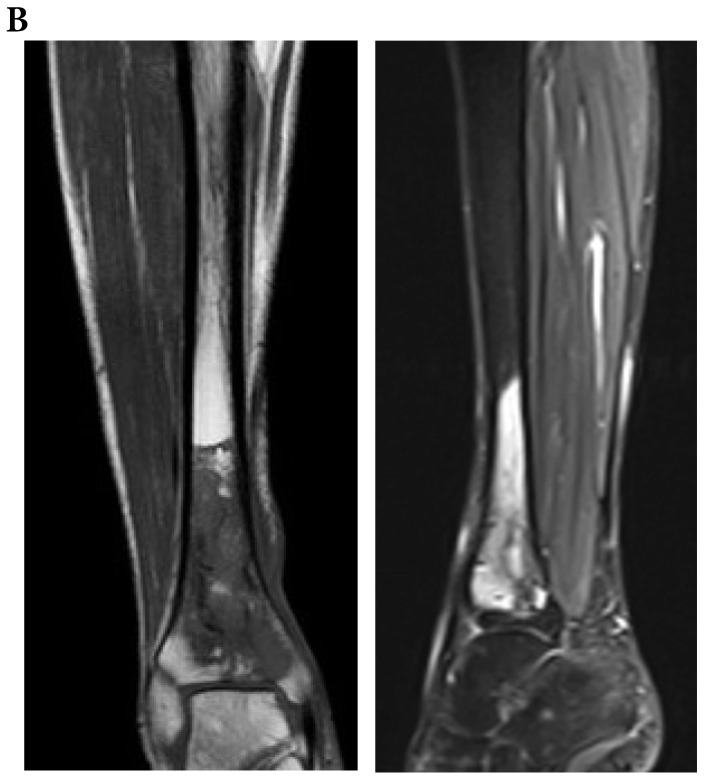
Pre-operative radiograph (a) and MRI (b) suggestive of Cierny and Mader Stage 3 osteomyelitis of the lower tibia.

At the time of our assessment he was afebrile and systemically well, though complained of ongoing ankle discomfort and swelling. On examination there was a scar over the anteromedial border of the tibia, with a small amount of skin discolouration and dry crust over the lower third. He had strong pedal pulses, normal sensation and a good range of ankle movement. Pre-operative imaging was consistent with Cierny and Mader Anatomic Type 3 osteomyelitis of the right lower tibia (Figure 1).

**Figure 2:**
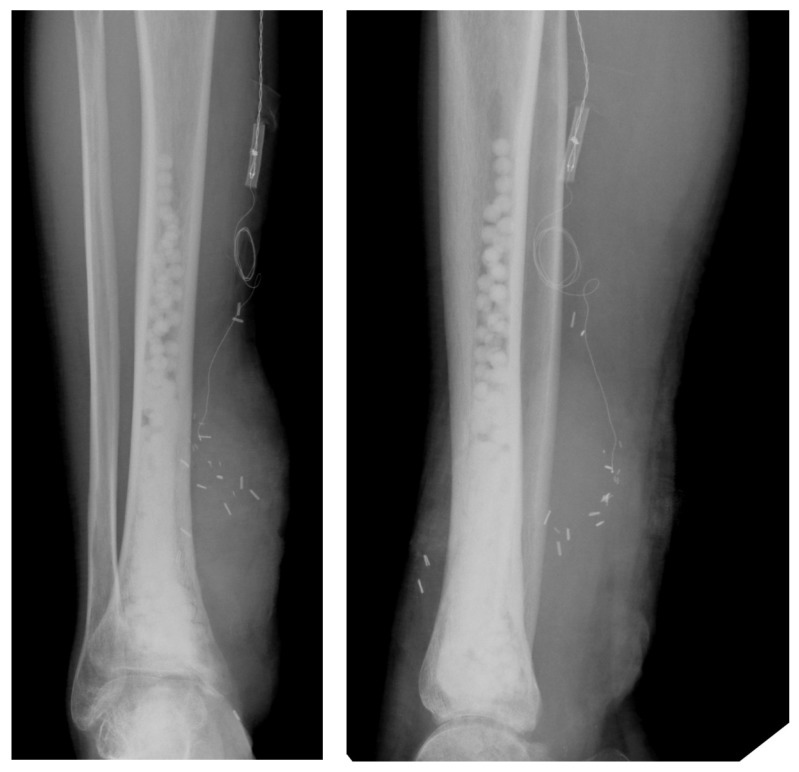
Post-operative radiographs with gentamicin eluting, bioabsorbable composites visible at the site of osteomyelitis excision.

He was taken to theatre for surgical excision, deep tissue sampling and reconstruction. The previous bone window was extended and the cavity was curetted back to healthy bleeding bone. Five deep samples were taken using a validated sampling protocol 31,32. Briefly this protocol recommends that when suspecting infection, 5 or more deep samples are taken with minimal manipulation of the target area using separate, unused instruments for each sample. Preferably samples should be taken prior to administration of intra-operative antibiotics, and after withholding antibiotics for at least 2 weeks prior to sampling. No pus was seen, but a significant area of dead infected bone was removed leaving a cortico-medullary bone defect. The resection site was washed with 0.05% aqueous chlorhexadine and the bone defect was filled with gentamicin eluting, bioabsorbable composites (Herafil® beads G [Heraeus Medical] and Cerament™ G (20mls) [Bone Support]). The soft-tissue defect was reconstructed with a free gracilis muscle flap and split skin graft (Figure 2). Post operatively he was treated empirically for five days with vancomycin 1g BD and meropenem 500mg TDS.

In the laboratory, a preparation from each deep tissue sample was inoculated into both a BD BACTEC™ Plus aerobic/F bottle and a BD BACTEC™ Lytic/10 Anaerobic/F bottle and incubated at 35-37 degrees within a BD BACTEC™ FX system. After 48 hours incubation *C. cadaveris* grew in four out of five samples incubated under anaerobic conditions (sensitive to penicillin, tetracycline, erythromycin, fusidic acid, rifampicin, linezolid, vancomycin and metronidazole, as assessed by disc diffusion). No other organisms (including further Staphylococci or Streptococci) were isolated. Deep tissue histology was consistent with active chronic infection, but no organisms were seen with Gram stain. He was commenced on a 3 month course of oral clindamycin 450mg TDS and discharged on post-operative day ten.

### Outcome and follow up

**Figure 3:**
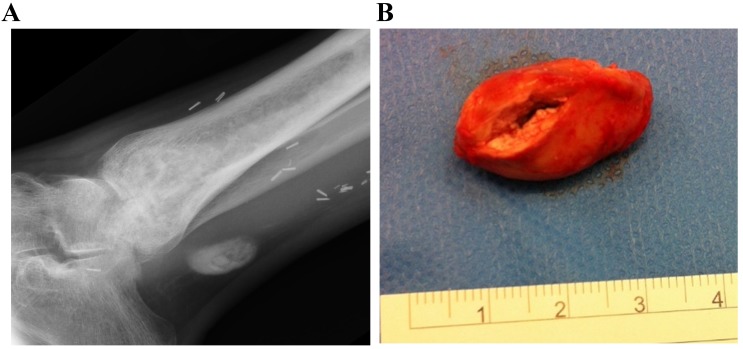
Radiograph (a) at 10 months showing a small radiodense area of extruded antibiotic carrier adjacent to the tibialis posterior tendon. The bone defect has remodelled well with no sign of recurrent infection. At operation, a 1x2x2.5cm soft mass of calcium sulphate was removed (b).

At follow up 6 weeks later the wound was healed and the patient was systemically well. At 9 months after surgery, he still complained of ongoing pain and stiffness behind the medial malleolus. This was due to irritation of his tibialis posterior tendon by a small collection of calcium sulphate extruded from his bone defect (Figure 3A). He underwent excision of this without complication (Figure 3B). He was discharged from our care 21 months after his initial surgery, fully mobile and with no evidence of ongoing osteomyelitis.

## Discussion

This is the first published case of *C. cadaveris* osteomyelitis. That this unusual pathogen was isolated only at the time of definitive surgery following multiple failed therapeutic strategies highlights the importance of robust deep tissue sampling in the management of chronic osteomyelitis. It is likely that this infection was caused by direct inoculation following a contaminated soft tissue injury. There were no other identifiable host risk factors. 

With all chronic infections it is essential to identify the causative organism to permit targeted antibiotic treatment, both to help prevent the development of multi-resistant pathogens and to contain infections in a timely manner. In this case, *C. cadaveris* had not been isolated from any samples taken prior to this most recent osteomyelitis excision. However, all of the cultures of deep wound samples taken previously had been inadequate: none had been cultured anaerobically. It is imperative, with chronic infections not responding to empirical or even targeted therapy, to ensure that appropriate cultures for fastidious organisms are completed. It is likely that *C. cadaveris* persisted despite the previous antibiotic courses due to incomplete excision as well as sub optimal duration of antibiotic therapy and poor penetrance of antibiotics to the site of infection.

Our decision to use a long course of clindamycin to treat this multi-sensitive pathogen was primarily based upon a concern regarding the possibility of an incomplete excision. Oral clindamycin has good bone penetrance and good activity against anaerobic bacteria. Concerns about pseudomembranous colitis are less immediate in a young, otherwise healthy man. Co-amoxiclav or metronidazole could have been suitable alternatives but the poor bone penetrance of oral beta lactams plus the side effect profile of metronidazole make these less favourable choices for a long term antibiotic course.

In summary, this case highlights the need for multiple deep tissue samples with appropriate culture to identify unusual causative organisms in chronic deep tissue infections. This is the first case of *C. cadaveris* osteomyelitis in the literature, identified only after suitable culture technique was employed. Identification of the causative pathogen enabled targeted antibiotic therapy which contributed to the overall successful outcome.

## Learning points

- *C. cadaveris* is an unusual anaerobic pathogen and previously unreported as a causative agent of osteomyelitis.
- Careful, fully sterile and repeated intra-operative sampling is essential for effective and targeted antibiotic treatment of chronic osteomyelitis.
- Anaerobic and aerobic cultures are essential to identify all relevant potentially pathogenic organisms.
- Antibiotic choice in chronic osteomyelitis should take into account extent of excision, organism sensitivities and antibiotic penetrance, mechanism of action and side effect profile, especially when long courses are indicated.

## Figures and Tables

**Table 1:** A summary of published cases of Clostridial osteomyelitis

Paper	Clostridium species	Type of infection	No. of cases	Case details *(where known)*
Mormeneo Bayo et al. 2020	Clostridium celerecrescens	Fracture related infection	1	39F sustained bilateral open femoral fractures in road traffic accident
Tremp et al. 2020	Clostridium spp.	Fracture related infection	1	43F farmer stumbled over doorstep, sustained trimalleolar open fracture
	Clostridium butyricum	Fracture related infection	1	58F fell into cesspool, sustained bimalleolar fracture and soft tissue injury
Vijayvargiya et al. 2019	Clostridium paraputrificum	Septic arthritis and osteomyelitis	1	86F native shoulder joint septic arthritis and osteomyelitis
Abusnina et al. 2019	Clostrodium sporogenes	Osteomyelitis	1	66F obese non-insulin dependent diabetic with pressure ulcer associated osteomyelitis
Hirai et al. 2017	Clostridium hydrogeniformans	Fracture related infection	1	18M sustained open right arm fracture in motorbike accident contaminated by drain contents
Perkins et al. 2017	Clostridium sphenoides	Fracture related infection	1	20M sustained open right radius and ulna fracture whilst wrestling, contaminated with soil.
Mutoh et al. 2015	Clostridium innocuum	Osteomyelitis	1	32M with ALL and pelvic osteomyelitis and bacteraemia, unclear source
Virot et al. 2015	Clostridium tertium	Osteomyelitis	1	40M ex soldier with osteomyelitis surrounding shrapnel in tibia
Ibnoulkhatib et al. 2012	Clostridium spp.	Fracture related infection	12	Multiple cases of traumatic fracture, each with soil contamination
Mischnik et al. 2011	Clostridium celerescens	Fracture related infection	1	55M with peripheral vascular disease and a history of open tibial fracture
Taylor et al. 2010	Clostridium argentinense	Fracture related infection	1	34M with contaminated open fracture of right ulna and radius playing soccer
Jiang et al. 2009	Clostridium glycolicum	Fracture related infection	1	20F with open fracture of radius and ulna after a road vehicle accident
Tekaya et al. 2008	Clostridium clostridiiforme	Vertebral osteomyelitis	1	
Kihiczak et al. 1999	Clostridium septicum	Osteomyelitis	1	
Shetty et al. 1998	Clostridium septicum	Osteomyelitis	1	
Scanlan et al. 1994	Clostridium bifermentans	Osteomyelitis	1	81M with oropharyngeal lymphoma, bacteraemia and multifocal osteomyelitis (T11-L5, sacrum and ribs) of unclear source
Spitzer et al. 1991	Clostridium clostridiiforme	Osteomyelitis	1	
Brook et al. 1993	Clostridium spp.	'Bone infection'	1	
Neimkin et al. 1985	Clostridium septicum	Osteomyelitis	1	

## Abbreviations

ALL: acute lymphocytic leukaemia
MALDI-TOF: Matrix-Assisted Laser Desorption/Ionization-Time of Flight
BD: bis die (twice daily)
TDS: ter die sumendum (three times daily)
QDS: quater die sumendus (four times daily)
GP: general practitioner
